# Implementing a new living concept for persons with dementia in long-term care: evaluation of a quality improvement process

**DOI:** 10.1186/s12913-024-10765-y

**Published:** 2024-03-07

**Authors:** Suzanne Portegijs, Adriana Petronella Anna van Beek, Lilian Huibertina Davida van Tuyl, Cordula Wagner

**Affiliations:** 1 Netherlands Institute for Health Services Research (Nivel), PO Box 1568, 3513 CR Utrecht, The Netherlands; 2 Viva! Zorggroep, Care Organisation, Parlevinkerstraat 23, 1951 AR Velsen-Noord, The Netherlands; 3 Department of Public and Occupational Health, Amsterdam Public Health Research Institute (APH), Amsterdam UMC, Vrije Universiteit Amsterdam, Van Der Boechorststraat 7, 1081 BT Amsterdam, The Netherlands

**Keywords:** Dementia, Long-term care, Autonomy, Ethnography, Mixed-methods, Quality improvement, Open environment, Process evaluation

## Abstract

**Background:**

Improving quality of nursing home care for residents is a constant focus of stakeholders involved within quality improvement projects. Though, achieving change in long-term care is challenging. Process evaluations provide insight into the nature, exposure and experiences of stakeholders and influencing mechanisms for implementation. The aim of this study is to gain insight into the process and facilitating and hindering mechanisms of implementing a quality improvement project that seeks to create a dementia-friendly community with a nursing home at its core.

**Methods:**

For the process evaluation we planned a case study design with an ethnographic approach. Various research methods were used: qualitative observations, focus groups, interviews and questionnaires for various stakeholders and document review. Data collection and analyses in this study is based on the Consolidated Framework for Implementation Research.

**Results:**

Four main lessons were learned. Firstly, nursing staff are crucial to achieve more freedom for residents. Secondly, high-impact changes in daily care need strong and sustainable focus from the care organisation. Thirdly, dementia-friendly societies should be deployed from multiple actors, which entails long-term collaborations with external stakeholders. Fourthly, the transition to a dementia-friendly society requires meeting spaces for and a focus on both residents and people from the community. Consequently, local residents are shifting from external to internal stakeholders, extending beyond the regular involvement of informal carers and volunteers within the nursing home.

**Conclusions:**

Nursing homes are part of the local community and provide opportunities to collaborate on a dementia-friendly society. However, the change that is required (promoting freedom, residents’ autonomy and the redesign of care processes) is complex and influenced by various mechanisms. Understanding these mechanisms can benefit other care organisations that strive to implement a similar initiative.

**Supplementary Information:**

The online version contains supplementary material available at 10.1186/s12913-024-10765-y.

## Introduction

Improving the quality of nursing home care for residents (with dementia) is a permanent focus of many stakeholders involved, such as persons with dementia and their relatives, nursing staff and policy makers. Therefore, attempts to implement innovative concepts are frequently pursued, and often structured and evaluated using the Plan Do Check Act cycle (PDCA). PDCA is a method that ensures a continuous cycle in quality management [1]. Despite numerous efforts, achieving sustainable quality improvement in long-term care is challenging. This is partially because interventions are not always transferable, as most interventions are effective in some settings but not in others. To design potentially successful quality improvement interventions, it is important to make use of a detailed breakdown of the implementation process of multiple successful and unsuccessful interventions [2].

There are various mechanisms that influence quality improvement initiatives on micro, meso and macro level. On macro level, changes in healthcare policy on a national and regional level influence the potential success of quality improvement projects, such as available budgets and restrictions in healthcare provision [3, 4]. Mechanisms on meso level include organisational aspects such as funding, logistic and infrastructure difficulties; and flexibility of the organisational culture. Furthermore, a high number of quality improvement projects at the same time is disadvantageous for successful implementation [5, 6]. Micro level mechanisms of influence include characteristics of care staff, e.g. turnoverrate, absenteeism, workload, education level, communication and support, and general attitudes towards change [5, 6]. Also, care needs of residents and attitudes and viewpoints of residents and their family members are important aspects to take into consideration [5–7].

Large quality improvement projects in long-term care are scarce [8], particularly in creating dementia-friendly communities. It is therefore valuable for both future research and clinical practice to gain insight into the relevant mechanisms in which quality improvement of the healthcare organisation relates to changes in the community of which the organisation is part.

A way to effectively evaluate quality improvement initiatives and the possibilities for implementation in other organisations is conducting a process evaluation. Process evaluations enable researchers and implementers to (1) describe the intervention in detail, (2) check actual exposure to the intervention, and (3) describe the experience of those exposed [2]. A theoretical framework suitable for process evaluations focusing on quality improvement in healthcare is the Consolidated Frame for Implementation Research (CFIR) framework [9], which has been used earlier to describe interventions in long-term care. The framework asks for a reconstruction of a complex reality, in which quality improvement is influenced by organisational and contextual mechanisms [9]. The added value of the CFIR [9] is its strong emphasis on the organisational level and comparison between the intervention as intended and eventually implemented [2]. Moreover, it provides an immediate insight into the outcome of the intervention and has sufficient focus on the individual level. This makes it a suitable framework to monitor and evaluate the process of quality improvement projects, especially regarding dementia-friendly initiatives [10].

Ethnography forms -since many years- an important method within healthcare research and focusses on human social action within its specific context [11]. According to Gertner et al. (2021), the use of ethnographic approaches are well accepted within implementation research and are regularly combined with theoretical frameworks in order to gain insight into interactions and context within implementation processes [12]. Also, ethnography focusses on context-specific mechanisms that give the opportunity to gain an in-depth understanding of the underlying change processes [13]. Methods used within ethnography are mixed and take place over a longer period of time, and contribute to reflexive interpretation of change processes in terms of how these shape and construct the organisation in its environment [13]. Making ethnography well suitable for the purpose of gaining an in-depth insight into process evaluations [12].

Persons with dementia in long-term care facilities have complex care needs that often derive from multiple health problems. Due to their health problems, the majority of persons with dementia living in long-term care facilities reside in closed units and are not allowed to move freely outside the facility (without supervision) [14–18]. This impacts their possibilities for social interaction and participation in the community, and limits their physical activity, consequently, leading to a lower quality of life [19–22]. In the Netherlands, as the organisation of care for persons with dementia takes place in closed units in long-term care facilities, they have largely disappeared from society. As a result, people in the community often do not know how to interact with persons with dementia and how to help them if necessary [23]. Due to this, a care organisation within the Netherlands wants to change the way their long-term care is organized by carrying out a large quality improvement project that focusses on improving the participation of persons with dementia within the community. In the next section we will describe this project.

### The quality improvement project: towards a dementia-friendly nursing home that is part of the local community

In this study, we evaluate a quality improvement project in the south of the Netherlands during the period 2018–2021, initiated by a care organisation. The aim of the project was to improve quality of care for persons with dementia by integrating one of their long-term care facilities into the community. The changes of the organisation focused on 1) social cohesion; 2) person-centred care, and 3) increasing quality of life. The project was carried out in a nursing home and focused on improving quality of care and participation of residents with dementia within the local community. This focus translated into four core elements that were aimed to be altered during the quality improvement project; 1) Construction of the building; 2) Grounds surrounding the building; 3) The use of the Chapel on these grounds; 4) Place in the community. By changing these elements, the care organisation aims to create a new long-term living concept that allows persons with dementia to live in large individual apartments among both persons with and without dementia and by creating an open long-term care environment in which residents can leave and enter the facility independently. Furthermore, the project aims to place persons with dementia ‘back in the heart of the community’ by stimulating interaction between community members and residents of the care facility. As this is the initial study that follows such a large change initiative towards the creation of a dementia-friendly community with a nursing home at its core, the findings of this study can provide valuable insights into the feasibility of such a project and the relevant influencing themes and mechanisms.

## Methods

### Aim of the study

The aim of this study is to gain insight into the process and facilitating and hindering mechanisms of implementing a quality improvement project that wants to create a dementia-friendly community with the nursing home at its core. Subsequently, this study reflects on the lessons learned and implications for future initiatives and clinical practice.

### Research questions

Through this study, efforts are undertaken to provide answers to the following research questions:

- What interactions and mechanisms influence changes in care processes and quality improvement in nursing homes aimed at dementia-friendly communities?
- What are the perspectives and experiences of the various stakeholders involved regarding the quality improvement project, changes in care processes and implementation process?

### Reporting guidelines

To ensure a full description of the methods of this study, the reporting guidelines for ethnographic approaches according to Gertner et al. (2021) were used [12]. Based on the Consolidated criteria for reporting qualitative research (COREQ) [24], but with additional emphasis on certain items within the three domains: research team and reflexivity, study design, and analyses and findings.

### Setting at the start of the quality improvement project

Below we will describe the setting of the nursing home at the start of the quality improvement project according to the four core elements: 1) Construction of the building; 2) Grounds surrounding the building; 3) the use of the Chapel on these grounds; 4) Place in the community.

### Construction of the building

The nursing home was formerly a residential care setting for older persons and build in the 1970’s. Due to the healthcare reform in 2015 [3, 4] it officially became a nursing home. The nursing home is build on the location of a former seminary; this accounts for the large grounds and chapel that accompany the building. Over the years a temporary building for persons with dementia was build on the grounds, providing psychogeriatric nursing home care in a closed setting, see Additional file 1 for a description.

### Grounds surrounding the building

The grounds surrounding the temporary building were poorly maintained and consisted of dated allotment gardens and a field with horses. The grounds also contained a small cemetery for priests, a small lane consisting of trees that was used for prayer and housed a bee keeper. Additionally, the nursing home had a strong focus on social entrepreneurship and had multiple ties with local businesses and associations, including (among others) the butcher, baker, flowershop, the local youth club, and the allotment gardeners. Many residents of the community conducted voluntary tasks within the nursing home.

### The use of the Chapel on these grounds

The grounds also included a Catholic chapel build in the 1930’s. The chapel had a rectangular shape, stone floors and two rows of wooden church benches leading up to the altar. The church was still fully operational and used for religious services and funerals. On a local website more information about the history of the chapel is provided. Formerly, the chapel was part of a seminary that was used to educate boys to become missionaries.

### Place in the community

The nursing home already had multiple ties with the community, for instance, many residents with dementia lived within the community before admission. Also, nursing staff live in close proximity of the nursing home, either in the community or nearby villages. Several community activities were organised in collaboration with the nursing home.

### Research team and reflexivity

The research team consisted of SP, AvB, LvT and CW. Author SP has a background as a physiotherapist and currently works as a quality advisor and researcher within long-term care. AvB has a background in socioliogy, extensive experience as a researcher within long-term care and ethnographic research, and has a job function in both policy and research. LvT has wide experience within the field of research and has worked on numerous research projects with both qualitative and quantitative natures. CW is a professior in patient safety.

At the start of the innovation, a collaboration agreement was formed with the care organisation, Alzheimer Netherlands, ActiZ (sector association) and two scientific institutes, including Nivel, the Netherlands Institute for Health Services Research. As a result, researchers SP and AvB had prior encounters with stakeholders within the care organisation before the start of the study. Due to their (practical) experiences within the long-term care setting, both SP and AvB coordinated and conducted the various research methods. Though, to ensure as much objectivity as possible, LvT and CW had hardly any involvement with the collaboration partners and did not take part in the data collection.

### Study design

We made the decision for a case study design due to the complex and multidimensional approach in this quality improvement project. Our design is in line with the approach of Stake [25]. Within this study, an ethnographic (open holistic) approach is used combined with the CFIR [9] to structure data collection, thematic identification and outcomes from an extensive set of research methods, *see *Table 1.

**Table 1:** Overview of focus groups and interviews and specifications

Participant group	Research method	Inclusion/exclusion criteria	Participants	Focus	Important (decisional) information
Nursing staff	Focus group 1 *September 2019 Focus group 2 *October 2021 *Both focus groups lasted 90 min*	All nursing staff of the nursing home were eligible for inclusion and recruited through the manager of the nursing home	Focus group 1 *Certified nursing assistents (*n* = 2) *Age = 33 en 49 years *Working experience in care = 16 and 30 *All female Focus group 2 *Certified nursing assistants (*n* = 4), care assistants (*n* = 2) and a recreational therapist (*n* = 1) *Age = 49–65 *Working experience in care = 3-34years *All female	Focus group 1 *Quality improvement project *Perspectives on autonomy and freedom See Additional File 2 for the interview guide **Part of larger research project, see earlier publication [7] Focus group 2 *Quality improvement project from the ‘individuals involved’-perspective of the CFIR *Questions derived from earlier collected data See Additional File 2 for interview guide	The research team specifically asked for a mix in job function, experience in care and years of employment at the nursing home to ensure an accurate reflection of the entire group of nursing staff was invited
Nursing staff	Questionnaires *June 2018 *May 2021	All nursing staff working at the nursing home were eligible for inclusion	2018 *n = 18 2021 *n = 14	*Perspective on long-term dementia care and quality improvement project *Social connections with the local community *Attitude towards persons with dementia	The questionnaires were placed in the office of the nursing staff. It is therefore unknown how many questionnaires were handed out
Management	Three focus groups *October 2019 *March 2021 *November 2021 *All focus groups lasted 2 h*	Participants were only eligible for inclusion if they had a position in management within the care organisation/nursing home	4 to 6 participants with either a policy, management or director position	An interview guide was used based on the five domains of the CFIR [9] and information retrieved from other research methods See Additional File 3	-
Family members	Three online and telephone interviews in November 2021 *All interviews lasted 30 min*	All family members of nursing home residents with dementia were eligible for inclusion and invited through a letter	*Spouse (*n* = 1) *Child (*n* = 1) *Informal caregiver (n = 1) All participants were connected to different residents	*Experiences with and attitudes towards the changes *The implications of the changes for the residents *The implications of the merge for the (intended) changes	The response was too low to organise a focus group. To ensure an informal setting, but at the same time describe the information provided accurately, summaries were made directly after the interviews, including the field notes of the researcher
Family members	Questionnaires *May 2018 *October 2021	“ “	2018 *9/56 2021 *16/50	*Health of their relative *Social connections with nursing staff *Perspective on the quality improvement project **Regarding social connections: part of larger research project, publication currently under review	-
Residents	Six ‘Go-along’ interviews *June 2018 (2 interviews) *October 2021 (4 interviews) *Interviews took 45–60 min*	*All residents with dementia that lived in the nursing home were eligible for inclusion *Written consent from family members *Interview only took place when residents agreed upon invitation	*1 male *5 female	To gain insight into how residents experience their environment and whether they see a connection with their own personal life	*A form of in-depth qualitative research to explore the experiences of persons with their familiar environment [26]. *As persons with dementia often express extensive forms of non-verbal communication, summaries of the interviews were made instead of recordings, including field notes and observations
Community members *June 2018 *October 2021	Questionnaires *June 2018 *October 2021	Community members that live in close proximity of the nursing home	2018 *141/250 2021 *63/340	*Social connections of community members with residents and staff *Frequency of visits to the nursing home *Perspective on the quality improvement project **Regarding social connections: part of larger research project, publication currently under review	The questionnaire for the community members was developed in cooperation with the nursing home

#### CFIR

The Consolidated Framework for Implementation Research [9], distinguishes five domains: 1) The ‘Intervention Unadapted’ is the intervention as it is originally intended and the ‘Intervention Adapted’ as eventually realised, both consisting of the core components and an adaptable periphery. 2) The ‘Inner Setting’ focusses on the structural, political and cultural context within an organisation. 3) The ‘Outer Setting’ represents the financial, political and social context. 4) The fourth domain ‘Individuals Involved’ includes the individuals that are both actively and passively involved in the intervention and/or implementation process. 5) The fifth domain ‘Process’ refers to the active change process that consists of multiple subprocesses. [9].

### Ethnographic approach

To address the complexity of the quality improvement intervention central within this study, the implementation process was studied from an ethnographic point of view using thick ﻿description﻿. ‘Thick description’ is an essential part of an ethnographic approach, referring to the description of human social action [11] and is not limited to physical behaviours. It also comprehends the context in which these behaviours take place [11]. Leslie et al. (2014) have adapted the approach of thick description for research into healthcare quality improvement, where it refers to the aim of investigating how contextual and organisational mechanisms contribute to changes in professional behaviour, organisational culture and inter-organisational networks [13]. As this study comprehends a large change initiative that takes place over a long period of time, involves various stakeholders, is setting-specific, and requires a change in organisation of care and culture and involves the network surrounding the nursing home, we chose ethnography as our research method. The research method provides the opportunity to fully grasp the underlying mechanisms and interactions that are essential to achieve the intended change [12].

### Data collection

Data was collected during different points in time over the course of the study, *see timeline in *Fig. 2. The methods include observations, focus groups discussions with nursing staff and management, interviews with family members and residents, questionnaires conducted by nursing staff, family members and community members, and document analyses. The various data collection methods are described in more detail below.

#### Observations

Observations were conducted during separate time periods (May–July 2018, May 2019, and May and October 2021), *see timeline *Fig. 2. Various types of observations were conducted, including both ethnographic observations and observations using a predetermined observation list. During observations, the researcher aimed to make themselves as ‘invisible as possible’ to ensure representivity of the usual course of events. Nursing staff and family members of residents were notified that the researchers would be present and that they follow the implementation process. Though, nursing staff were not informed about the specific aim and focus of the study.

### Ethnographic observations

Observations were conducted in May–July 2018, May 2019, and May and October 2021. The observations were conducted within different settings of the nursing home and surrounding grounds including the wards, shared rooms, hall ways, restaurant (the chapel) and the park area. The aim of these observations was to gain a holistic view of the new long-term living concept and the accompanying new organisation of care, the social interactions of nursing staff and residents and their daily (working) life. Likewise, it gave the researchers the opportunity to closely follow the intended changes. Field notes were taken and expanded soon after into extensive and detailed reports. These reports included among others information about the location, atmosphere, individuals present and their interactions and conversations. Findings were discussed regularly within the research team to interpret the findings, provide context and determine whether a follow-up was necessary. During the observations, the researchers also conducted small informal interviews with nursing staff, residents, family members and community members to gain insight into their perspective and experiences regarding the new long-term living concept and physical surroundings.

### Predetermined observations lists

The predetermined observation list (32-items) was used during observations on the wards and included items about ambiance, safety of residents, social interaction and physical activity. The observation list was derived from the aspects of quality of care defined by Rantz et al. (1998) [27, 28]. Items were scored on a five-point scale. The observations were on three separate moments during the morning, mid-day, and afternoon. All observations were carried out by two researchers simultaneously and discussed directly after. The results of these observations have been published in an earlier study, part of the larger research project [15].

#### Focus groups, interviews and questionnaires

Various focus groups and interviews were held with nursing staff, management, family members and residents, see Table 1  for an overview*.* All focus groups were recorded and transcribed verbatim.

In addition to the three focus groups with the management team, an online informal conversation took place with the project leader in January 2021 about the shift in job function, the corporate merge, COVID-19, and the organisation of care within the new living concept. A summary of the interview was made directly after by the two involved researchers.

#### Relevant documents

Various documents were received from the care organisation, including minutes of project group meetings, strategic orientations and vision documents. Moreover, several documents were retrieved from other sources, such as the internet and local news papers containing publically available quality reports, videos, vision documents and news items. In the context of privacy, direct reference is not always made within the results section. As an alternative we indicate from which type of source the information is retrieved. Documents on the Dutch Healthcare System were also used.

#### Quotes used from various data sources

All quotes were translated using freely available translation software and checked by a bilingual speaker.

### Analyses

**Fig. 1:**
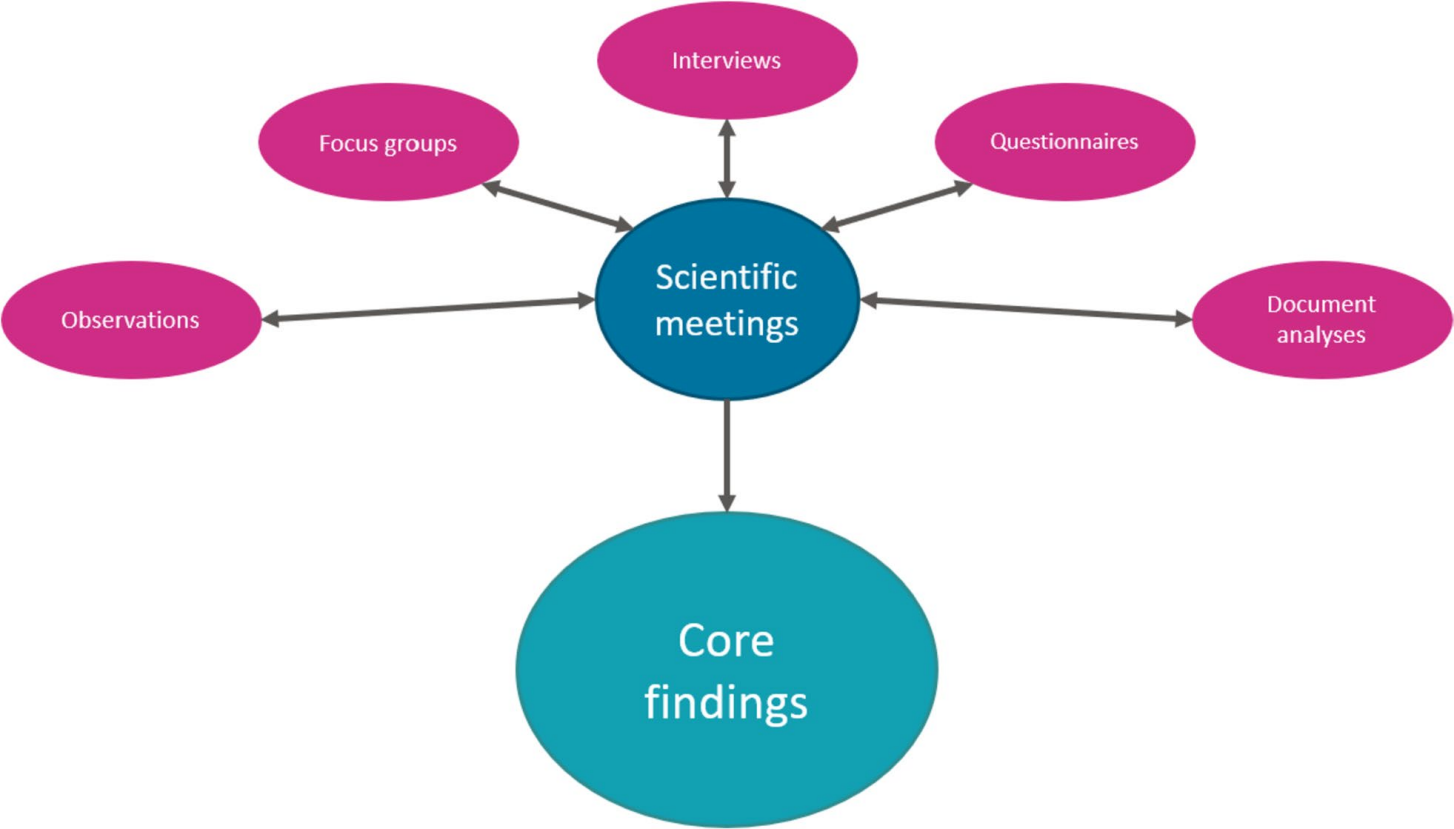
Analyses process of ethnographic approach using different research methods

In order to derive the core findings from the research methods used and ensure an iterative process, the research team held regular scientific meetings over the multi-year course of the quality improvement project. Within these meetings, the research methods, thematic identification (using CFIR) and outcomes were discussed and reflected upon. Scientific meetings were increased during data collection weeks. When needed, the approach for data collection and/or research methods were altered, see Fig. 1. For instance, over the course of the project it became apparent that the information from the focus groups with the management team were largely one-sided and slightly rosier than observed by the researchers. As a result, the emphasis on other research methods, especially the ethnographic observations, became stronger.

As described under ‘Research team and Reflexivity’, to borrow sufficient objectivity, LvT and CW were hardly involved in the data collection and communication with the care organisation. Using an inductive approach, the continuous discussion of and reflection on the identified themes and outcomes by the research team subsequently resulted into the core findings of this study. These core findings, were reported back to the care organisation in May 2022 through a consortium meeting. Multiple persons from the management team that were also involved in the focus groups were present, alongside different other involved parties such as representatives from a collaborating university and national branche organisation. The core findings were discussed and reflected upon by all parties present. As the care organisation acknowledged the core findings and agreed with the results of this study, no alterations were made.

## Results

The care organisation that is followed within this study aims to implement a new long-term living concept and bring long-term care back in the heart of the community by altering four core elements: 1) Construction of the building; 2) Grounds surrounding the building; and 3) The use of the Chapel on these grounds 4) Place in the community. In the following sections the results will be structured by the five domains of the CFIR [9]. Firstly, the changes as initially planned (‘Intervention unadapted’) and eventually realised (‘Intervention adapted’) are presented, see also Table 2. We then describe the results divided in process, inner setting, and outer setting.

**Table 2:** Overview of ‘Intervention unadapted’ and ‘Intervention Adapted’

Core element	Intervention unadapted	Intervention adapted
Construction of the building	As determined prior to the start of the study, the nursing home was going to be structurally reorganised in multiple ways First, the old building (± 25 years old) was to be demolished and rebuild into 150 individual apartments in several buildings. Part of these buildings were going to be realised by the housing corporation. In-house sensors, innovative wearable sensor technology were going to be installed in all buildings as part of usual care to ensure the safety of older persons with dementia. In the new buildings, persons with dementia and persons without dementia will be living together. Older persons from the local community could live in one of the apartments even when no care was required or when they wanted to accompany a spouse with dementia that is admitted to the nursing home. Furthermore, an open long-term care environment was to be created that gives all residents the opportunity to leave the building independently en roam freely on the surrounding grounds and neighbourhood. Residents would uphold their own individualised daily schedule, group activities would be optional.	The new nursing home, that was part of the rebuild, has been opened in November 2020 and currently provides long-term care for residents with and without dementia. Residents live in large spacious individual apartments with innovative technology. The old building is not yet demolished and the building of the individual apartments for persons -with and without care- on the grounds has not started. The open long-term care environment has been realised, meaning that all doors are open for residents and they have the freedom to leave the facility independently and roam around the park and surrounding neighbourhood. Despite the original plan to let persons with and without dementia live next to and amongst each other, these groups are currently clustered on separate floors. This limits the social interaction between residents with different care demands. Persons living in the nursing home without dementia have the opportunity to choose their own individual daily schedule. Yet, the persons with dementia spend the majority of their day in shared living rooms that uphold a daily time schedule and structure, mostly focusing on group activities.
Grounds surrounding the building	The facility grounds with an area of 6 hectares, were going to be restructured into a green park to make it attractive for residents and people from the surrounding neighbourhoods. The park area aimed to provide meeting opportunities for residents with dementia and people from the local community through the realisation of broad walking paths, benches, fishing pond, playground for children and a day centre for community members in need of care. Moreover, the existing allotment gardens would be renewed and the bee keeper -already on the grounds- would be preserved. The municipality had decided that some elements should be retained. These included the cemetry for former priests and the old lane of trees, that was used for prayer. The vegetation of the park would be improved by placing many bushes, plants, greenery and large old trees The park area was to have no external barriers and be freely accessible for all to stimulate interaction between residents from the nursing home and people from the local community.	The surrounding grounds have been turned into a large park-like area with new allotment gardens and green house, a fishing pond, walking lanes and a few benches for visitors to sit. The bee keeper, cemetery and trees on the grounds are preserved in accordance with the plans. However, there is limited additional vegetation and not all areas of the park and walking paths are accessible for persons with physical disabilities. A very small number of residents leave the facility independently and visit the park area. The interaction between nursing home residents and community members in the park is limited.
The use of the Chapel on these grounds	The chapel was to be rebuild into a restaurant for both residents with dementia and persons from the local community. The restaurant aimed to have a terrace, employ a permanent chef and serve both lunch and dinner. The chapel is to be visible from all corners of the grounds and serve as a central meeting place for both residents and people from the community.	The chapel has been renovated and was turned into a restaurant that opened in March 2019. The chapel serves both lunch and dinner for residents and community members, has an open terrace near the front door of the new nursing home and employs a permanent chef Residents visit the restaurant often with family members and visitors. Also the care organisation uses the restaurant regularly for smaller and larger meetings with external partners and nursing staff and large events. The care organisation has created multiple ties with local associations that repeatedly use the restaurant for their meetings and rehearsals.
Place in the community	In the initial stage, the care organisation had a strong focus on social ties with the community and social entrepeneurship. As the facility is situated within a small rural village, there is a strong social cohesion within the community resulting in extensive involvement of volunteers and mutual relationships between residents and people in the community. The care organisation wanted to broaden this social network and create a dementia-friendly community with the nursing home at its core, with a focus on well being for both residents and persons in the community.	The original involvement of volunteers and family members has been preserved and multiple collaborations with local entrepreneurs are still in place. Yet, the involvement of the community is not intensified and the aim towards a dementia-friendly society has been realigned.

### Intervention unadapted and adapted

The initially planned changes (Intervention Unadapted) and the changes eventually realised (Intervention Adapted) are illustrated in detail in Table 2. The findings are described according to the four core elements of the quality improvement project.

The comparison between the ‘Intervention Adapted and Unadapted’ shows that not all aims have been realised. When we look closer into the different events and aspects, we can dissect several reasons for this. We will discuss these results in the following sections, structured by the CFIR-domains ‘Process’, ‘Inner Setting’ and ‘Outer Setting’. An overview of the main findings per domain is specified in Table 3.

**Table 3:** Overview of main findings per CFIR-domain

CFIR-domain	Main findings
Process	*Working culture of nursing staff
	*The commitment of participants towards the change that the project aimed to achieve
	*Changing role of community (members)
Inner Setting	*The corporate merge with another long-term care organisation and its consequences
Outer Setting	*Collaboration with external stakeholders
	*Influence of the COVID-19 pandemic

### Process

When we look at the results from our study three important mechanisms that influenced the quality improvement project emerge: 1) Working culture of nursing staff, 2) commitment of participants towards the change that the project aimed to achieve, and 3) Changing role of community (members).

Management name the working culture of nursing staff as a hindering factor in the project.

*“Because it is just very difficult, you know. If you start explaining it in theory, theory does not really catch on with the employees on our work floor. Because they are not used to thinking in theoretical concepts. That is not their job at all. That is not what they chose at all. That is not what makes them happy. […] You have to give employees tools and knowledge about dementia, because some really do not have that enough. But you also have to take that doom thinking away a bit: “but what if someone”. […] Yes someone can indeed fall anywhere and that can also happen in the park.”*

(Focus group with management, October 2019)

#### Working culture of nursing staff

The shift in working culture was seen as highly complex and the involvement of nursing staff and their guidance towards a new way of working was found crucial by the care organisation. The new care concept requires extra or more complex care tasks for nursing staff, such as monitoring basic care needs of residents while they are not in close proximity.

When we look at the results from the nursing staff themselves, it is clear that they had doubts from the start about the building, the open living environment for people with dementia and the new care concept. These doubts hinder the implementation process and are still evident at the end of the project.

*“I am very curious whether we can protect the safety of the residents in the open care environment.”*

(Questionnaire for nursing staff, June 2018)

*“Yes, but if someone has already left. I think to myself: I find that scary. That scares me. Because there's a pond over there. Yes, they say: people don't walk in the water. Yeah, I'm not so sure. When it's dark and they don't see anything."*

(Focus group with nursing staff, October 2021)

In our observations we find that nursing staff tend to immediately follow residents when they leave the floor or the building and subsequently limit residents’ ability to move around, see Table 4. This tendency reflects the absence of the culture change within the care teams that is essential to realize the necessary shift in the organisation of care.

**Table 4:** Qualitative observations conducted by the researchers at one of the living rooms in May 2021

A living room attendant, a care worker and 8 residents are present in the meeting room, spread across 3 tables. Sitting at one of the tables is a male resident with advanced dementia. In front of him is a plate with 2 half-eaten sandwiches. The living room attendant walks over to Mr. “Would you like some coffee?” She removes the plate and puts the uneaten sandwich in the hand of Mr. Then goes to the kitchen to make coffee. Puts milk and sugar in it, stirs it and brings it to the resident and puts it on the table
At another table sits a male resident in a transport wheelchair. I have not yet seen this resident sitting in a wheelchair. The resident prepares to get up. “Sir (….), please sit down for a while, dear”, says the living room assistant. She then sits down opposite the resident and has a chat. The resident again prepares to stand. “Lord (….), You have to sit in the chair for a while, because you are not feeling well. After eating a sandwich, you can go to bed. I think you want to go to bed, don't you? Yes, you are really not feeling well. Just wait until after dinner. I made delicious chicken soup.”
A resident at one of the other tables stands up. “(first name resident) will you stay seated? We're going to eat soon," says the living room supervisor. The resident walks towards the door of the meeting room. The living room attendant stands up and stands in front of her and asks: “Dear, where are we going? Will you help me with the soup?" The living room supervisor and the resident then have a conversation about chicken soup; whether vermicelli belongs in there or not and she takes the resident to the kitchen unit

#### Commitment of participants towards the change that the project aimed to achieve

Nursing staff indicate that their involvement in the change process was too limited. Hence, their commitment to the changes was also limited, which is not beneficial for the intended outcome. The involvement of nursing staff was initially characterized by face-to-face meetings and collaboration projects initiated by the care organisation. These were not continued during the project. Within the focus groups with management multiple reasons are mentioned, in particular the COVID-19 pandemic.

*“And then of course the lockdown came on March 15*^*th*^*. So the actual plan, the timeline that we prepared, we had to revise it a bit. So the preparation for the move and the approach that involved exchange days, shadowing days, meetings as a team. All sorts of things that, especially in that first wave, when digital working was not as common as it is now, we also genuinely did not know what was possible and what was not possible. And we were very careful, especially in our sector, because it was already clear at that time that our residents are the most vulnerable.[….]”*

(Focus group on management, March 2021)

In the results it becomes evident that nursing staff find it the responsibility of management to organize the involvement of nursing staff within the project. At the same time, the management of the organisation indicates that the commitment of nursing staff to the changes was limited. These contradicting views are illustrated in the following citations from a staff member and a member of management.

*“I feel they should ask… “Gosh, how are you doing? Just like with you now, like: “Gosh how do you experience the workload? Is everything still working out for you? I also think that it should come from upper management.”*

(Focus group with nursing staff, October 2021)

*“[…] But I think what is still needed, of course, and that is one of the important things we focus on in those six working groups, is that there really is a lot that has to be changed in these heads of the employees.”*

(Focus group with management, October 2019)

In addion, the results show that commitment of family members and persons in the community during the project was also limited, which is highly important in order to achieve successful change. From the beginning they have concerns about the safety of residents, which are not taken away during the project. At the start of the project, there was frequent communication with family members and members of the local community. However, during the course of the project, this communication came to a halt. According to management, this was again due to the COVID-19 pandemic. The lack of contact resulted into uncertainty for family members about the safety of their relatives and doubts about the care concept in general. Community members mentioned the lack of follow-up and their limited involvement in the changes that were made.

“Everyone who was involved is either somewhere else or gone. […] They don’t ask us anything […]. The idea is good, but the follow-up is not.“

(Owner allotment garden, October 2021)

#### Changing role of community (members)

Part of the change process was also to create a new role and place for the nursing home within the community, in order to work towards a dementia-friendly community. This included a focus on family and community members as part of the care concept. The aim was that residents with dementia were free and stimulated to walk into the village so that they could meet other persons. The results show that this aim is only partially realised. Residents are indeed able to walk outside the nursing home, which is facilitating for their participation within the community. Conversely, walking into the village is difficult as the old building still stands and forms a boundary between the new nursing home and the village. As a result, the park is also not so easily reached by persons in the community and limitedly used, which hinders the social interaction between community members and residents.

The results also show that community members and family members use the restaurant in the former chapel regularly, which is highly beneficial for the stimulation of social interaction and subsequent creation of a dementia-friendly environment. At the start of the project there were already many connections between the nursing home and persons and organisations in the community. These connections are also evident at the end of the project and therefore facilitating for the intended outcome of the project, see Table 5.

**Table 5:** Summary of walk-along interview with resident with dementia in October 2021

Mrs. points to the vegetable gardens and says that they used to come here often. I mention that the park has changed a bit lately. Mrs. confirms: 'Yes, definitely. Some say it will get even better. I hope I will still be here to experience that.' When I ask her what she thinks of it now, she says: 'I like it already.' We talk about needlework again. Mrs. says that she once made a scarf for the father. When asked whether the lady ever comes to The Chapel, she says that some people eat there in the evening, but the lady does not. ‘It is very cozy inside and we eat with a whole club. I like it very much. I like living here. I like to participate in things and have a chat.' Mrs. used to go to church on Sunday. She still does this sometimes, her children pick her up afterwards
We walk back to the entrance via the gravel path. The lady says that her children do not allow her to walk alone: ​​'I am not allowed to walk alone. I might fall. Someone always has to go with you.' Mrs. says she sometimes walks in the park and points to the gravel path. We continue along the path past the entrance of The Chapel. Mrs. looks at her feet: ' It's also difficult to walk here, isn't it, for us it is. There's all sorts of things on the floor.' Mrs. points to the twigs and acorns on the floor
When we enter the location we meet the location manager. We say hello. The location manager points to the knitting and says she really likes it. Then the doctor arrives. Mrs. exclaims enthusiastically: "Ah, doctor van T. Will you come and visit me for a while?" The doctor says that he wanted to drop by, but that the door was locked and that he has to go now. A healthcare worker sees the doctor and speaks to him. They walk down the hallway together. When I walk with the lady towards her room, we pass the doctor again. Then she asks: "Hey doctor van T, did you come to see me?" The doctor again replies that he came by but that the door was locked. "I'll come again next time. Then we'll have a cup of coffee together

The quality improvement initiative aimed to transfer community members from the external social network of the care organisation to internal stakeholders within the new concept. Though, it is evident that this is not realised at the end of the project while it is crucial for successful implementation of the new care concept. This is also illustrated in the way in which management speak about persons in the community at the end of the project. In the next section we will discuss the reasons for this change in orientation.

*“ […] That community concept in which, we are there for everyone and everyone is welcome, and everyone can join here. We no longer have the capacity for that at this time.”*

(Focus group with management, November 2021)

### Inner setting

The focus of this section is the corporate merge with another long-term care organisation and its consequences.

During the course of the project a corporate merge took place in July 2019 with another long-term care organisation in the region. Multiple reasons for the merge were given by the organisation, including financial benefits, more diverse care provision, more opportunities for innovation and mutual learning from each other’s strengths.

**Fig. 2:**
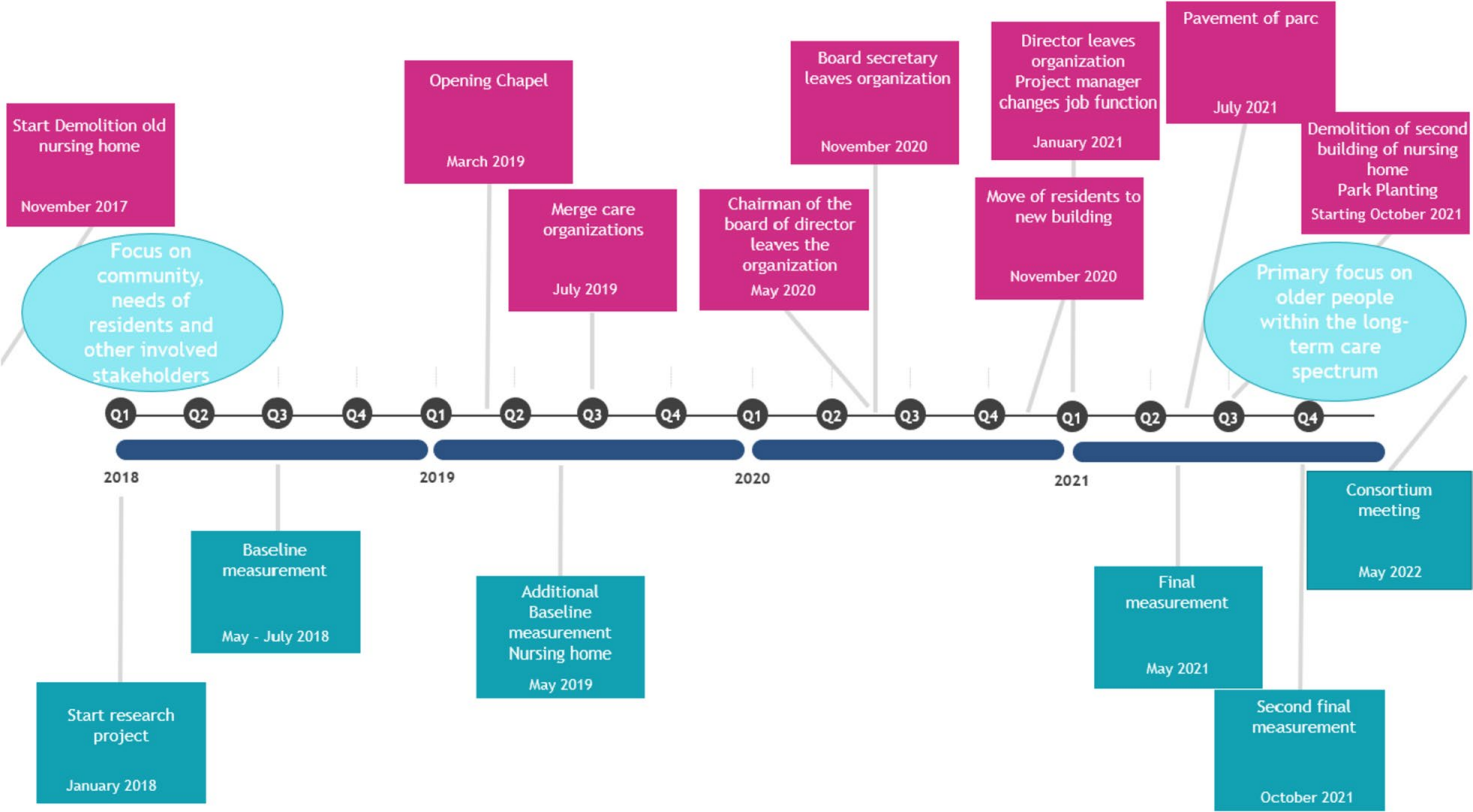
Timeline of the project with the most important milestones for the organisation (pink) and the research team (blue)

This corporate merge has been a major hindering event within the implementation process. Almost all employees on the administrative and management level that were directly involved in the development and execution of the quality improvement project received a new job function or left the organisation, including the chairman of the board of directors, the director of care, the project manager and the secretary of the board, see timeline in Fig. 2. As a result, the advocates for the quality improvement project disappeared.

The merge also affected the organisation of daily care, including the introduction of new care teams and new job profiles. After the merge, a clear distinction was made between care-related tasks and (leisure) activities that benefit the well-being of the residents. Many staff members that were originally involved within the project left the organisation or were reassigned.

*“But it is true, all the people involved and you mention [several names that have been discussed before] but then I would like to also mention the project team that was involved is gone. The last one will leave in a moment. She has her last day at work today. The collegiate team will then cease to exist.”*

(Focus group with management, March 2021)

This resulted into new employees being involved in the implementation (but not the initiation) of the quality improvement project. The remaining original employees – management and nursing staff- struggled with the changes, and as the new employees raised new ideas that did not always suit the original plans, it created the perception that ownership was lost.

*“This is a nice project, trajectory, also for the people who are closely involved, they also think wow, who will be joining the trajectory now and what are their ideas?” [red: sarcasticly spoken]*

(Focus group with management, March 2021)

The results show a shift in the vision regarding the quality improvement project. Initially, the care organisation focused on interaction with the community. This can be described as a community-driven approach where residents and community members are all part of the dementia-friendly community, focusing on the well being of all*,* see Table 6. An open environment for persons with dementia and the new building -with living appartments for residents with and without dementia- were aspects to achieve this aim. During the project we see a shift in this focus. The open environment for persons with dementia and the new building became aims in itself. This results in residents moving to other care locations when their care needs (either perceived by family members or care staff) do not fit the open environment and living arrangements of the facility.

*Basically, our general principle is 'no, unless'. That's just how we do it. We see domotics as an intervention that is part of the care and treatment plan. Not as a basic facility. That is already a very important point of view. Very different from how it is sometimes used at other facilities. If the behaviour of the resident is so extreme that you want to intervene, that is not possible in our organisation. We do have solutions elsewhere, but that will not happen here. We think that often it leads to restrictions for other residents.*

(Focus group with management, October 2019)

**Table 6:** Shift in focus within care organisation during the course of the project

Focus of care organisation in 2018	Merger statement November 2018	Focus of care organisation in 2021
*“[Name care *organisation*] not only provides care to its clients, but also sees it as its mission to bring together people’s desires and needs. The care demand of the clients and the needs of all those involved in the environment are guiding principles. We think and act from the point of view of the community* *That is more than just the teams and the clients. The community, that's what we are together:* *our locations and all those involved around them* *When speaking about our locations, we tend to call it communities. […]* *Enjoyment, a twinkle in the eye, feeling at home and belonging. Those are important valuses in [name care* organisation*]’s vision. [Name care organisation] wants to become more at the heart of the community with its various locations. A central place in a village or *neighbourhood*, where people can meet and enjoy.”*	*“An important starting point for the collaboration is the preservation of the local customs, village traditions, so that people can continue their lives in their own familiar residential and living environment. A nice place in a village or neighbourhood where people enjoy themselves and can meet each other and where we value the enjoyment of life for clients and a twinkle in the eyes of clients, employees, volunteers and other stakeholders above anything else. The continuity and quality of care will of course be maintained and will even improve in a number of areas* *Both organisations embrace the concept of housing and living in freedom. With the new organisation, they have the ambition to also enable people with dementia to move freely within an open yet safe living environment.”*	*“[Name care organisation] is a solid, resilient and progressive care organisation for clients with complex care needs in [name of region]. Older people who need intensive care and support can count on us. We offer a nice and sheltered place to reside and live. But clients who still live at home with complex care needs, or people who need temporary care and support, can also rely on our care, expertise and reliable and expert employees* *We do not do that alone. Employees and volunteers work closely with care partners and the social network of the clients. Think of family, friends, acquaintances and neighbors. Together with that environment, we want to contribute to a valuable life for a client. Employees talk to clients and their environment to determine together what aspects could be a valuable contribution to this phase of someone's life. Clients keep control as much as possible. Because if you are vulnerable, you are also entitled to your own freedoms and wishes. You want to 'matter'; to do things independently for as long as possible*

In addition, the overall focus of the organisation shifted from community-based care to residential long-term (dementia) care. Family members and community members are no longer considered as main stakeholders by the care organisation, but mainly as informal caregivers for residents of the organisation. There is a new focus on care-related tasks, instead of wellbeing and individual activities for residents. At the end of the project separate ‘hospitality staff’ are in charge of supervision on the living rooms and leisure activities. This results in group-oriented activities and daily care that is provided in the living room in which the freedom of resident is limited. These internal group-oriented activities make participation of the community hardly possible. Joint activities are only realised in the chapel, while divesment of the restaurant has been considered by the care organisation.

### Outer setting

This section describes 1) the collaboration with (external) stakeholders, and 2) influence of the COVID-19 pandemic.

#### Collaboration with external stakeholders

From the start, multiple external stakeholders were involved in the quality improvement project, including an architect for the design of the new facility and church, the local church for the take-over of the chapel, a constructor to execute the build, the provincial council (to retrieve permits for the fishing pond), the domotica supplier, community members and the allotment garden association.

Another important external stakeholder within the quality improvement project was the municipality. From the beginning, they were very positive about the quality improvement project. The multi-year vision of the municipality state that they are aiming to become dementia-friendly and therefore embrace the initiative. Certain elements of the project relied on this intention of the municipality, such as the day centre for community members. Based on the municipality finance structure that was introduced in 2015, financing these day centres is a responsibility of municipalities [3, 4]. Despite willingness from both the care organisation and the municipality to realize these elements, the funds available turned out to be financially insufficient for the initial plans. Eventually resulting in reduced support from the municipality and cancellation of the day care centre, which had major negative consequences for the intended change.

Collaboration with the municipality was also needed for the build of the new buildings and to receive permits for among others the restaurant within the chapel. In order to build the new buildings, the construction traffic had to go through the park area and created nuisance for the neighbourhood residents. The permits for the Chapel took a long time to receive for the care organisation as its destination plan did not fit the plans for the restaurant.

Another important aspect regarding the involvement of external stakeholders is that some are not familiar with the long-term care setting, which is impeding and makes it challenging and complex to facilitate collaboration. Despite the extensive investment in these collaborations from the care organisation. The complexity became apparent within, among others, the collaboration with the technology supplier.

*“[….] the system offers us many possibilities and many things are going quite well, but we have also learned some things. For example that it is very important to really be on the same page with your domotics supplier and to look at things from the viewpoint of the user. We have come across a problem were some devices on paper look completely functional, but the reality is that something cannot be charged conveniently or you have to press a button for 3 seconds with a certain force that our residents simply do not have. You know, those are learning points.”*

(Focus group with management, March 2021)

#### Influence of COVID-19 pandemic

An important environmental factor that should be mentioned is the COVID-19 pandemic, which had a massive effect on the (long-term) care sector in the Netherlands and subsequently influenced the course of this new long-term living concept negatively. For instance regarding the move of the residents with dementia from the old building to the new. It was set to take place in the fall of 2020 in the midst of the second wave of the pandemic in the Netherlands. As multiple residents had COVID-19 during the move, all residents were moved as cohorts and placed on separate wards, which did not fit the new concept where residents were able to walk around freely, live among and interact with residents with different care demands. As a result, many residents with dementia live almost exclusively among other residents with dementia.

*[….] Because they moved as a cohort you miss that moment, that moment of moving that you wanted to seize from and say: okay now those doors are open. […] They really had the idea of: ​​we are going to mix it up right away and then we're going to incorporate them right away. And now you actually start with a delay and that was a shame. […] That was really due to COVID, that was so different from what we had thought of beforehand.*

(Focus group with management, October 2019)

Furthermore, the COVID-19 pandemic resulted into restricted access to the nursing home for multiple months in 2020. This is at odds with the initial intentions of the research project. Afterwards it was very difficult to restore the contact as COVID-19 created uncertainty within care organisations and a strong focus on many challenges within the inner setting such as the organisation of vaccinations.

## Discussion

The aim of this study was to gain insight into the process of implementing a quality improvement project that wants to create a dementia-friendly society around a nursing home. Additionally, this study reflects on the lessons learned and implications for future initiatives connecting institutionalised nursing home care with dementia-friendly communities. We structure the discussion section using the core findings of this study.

### Core finding 1 – Crucial role of nursing staff

*More freedom for persons with dementia to stimulate physical activity is crucial, however, simply creating more freedom is not enough to make a change. Nursing staff are key to provide freedom and a culture change from traditional nursing home care is essential. In order to achieve this, nursing staff have to be involved intensively within the change process and facilitate them to new ways of working. It is imperative that sufficient safety and comfort is offered by the care organisation during the transition process.*

This core finding corresponds mainly with the ‘Process’-domain of the CFIR, as it refers to the degree of involvement of nursing staff and the preconditions needed to let hem become familiar with the new care concept.

This project again shows that realizing change in long-term care organisations is challenging and is dependent of many themes and mechanisms [5, 6, 29, 30]. These findings are similar to other studies. In the study by van Haeften et al. (2015) [29], which focused on an initiative to increase the freedom and autonomy and person-centred care for persons with dementia in six nursing home-based day care centres in the Netherlands, it was found that the nursing staff on the micro level play a crucial role in enhancing freedom and autonomy. This is also supported by other studies [7, 16, 31]. Van Haeften et al. [29] experienced limited flexibility and ability to adopt the new way of working among the nursing staff involved, which impeded the implementation process. A finding that is similar to our study, as we noticed a consistently present fear of harm for residents among the nursing staff, which hindered them to adopt the new organisation of care. Fear of harm for residents with dementia when providing freedom is also found in other studies [7, 32, 33] and an imperative aspect to take into account when aiming to change the organisation of institutionalised long-term dementia care.

An important note that should be made is that within this quality improvement project, a working-group-structure was used to engage nursing staff within the change process. This structure might not be sufficient enough to realize a shift within the long-term care culture, essential for these types of innovations. Realising such large changes within the ‘core’ of the long-term care culture requires a safe and comfortable environment provided by the care organisation and policy that supports innovation and new care models [8, 30, 31, 34]. We found that this has not been provided within this project.

### Core finding 2 – Importance of management commitment

*Constructing a dementia-friendly society around a nursing home requires a clear focus from the nursing home and preparedness to let go of traditional nursing home care. This requires a long-term investment from people at all levels of the organisation, including financially. Long-term care organisations are regularly subject to changes due to internal organisational events and mechanisms *[6, 35] *(e.g. mergers), making it difficult to maintain the same focus over a longer period of time. In line with the reflection on the first core finding, it is therefore of great importance when implementing a large-scale innovation that personnel and management are commited and personnel changes are kept to a minimum.*

As this core findings mainly focusses on organisational events, such as mergers, it fits best within the ‘Inner Setting’-domain of the CFIR. Mergers in health care settings and their consequences are mainly an internal matter and influence all levels of the organisation.

It is vital that care organisations have stable management with a clear long-term vision and are willing to deploy additional resources (financial or staffing) to give nursing staff the opportunity to adjust to the new situation. [29, 36] This lacked within this quality improvement project due to mainly the corporate merge, the changing financial and political climate and to some extent to the COVID-19 pandemic.

Regarding sustainable funding, a major element was the municipal finance structure on which certain elements of the project were based, such as the day care centre for persons from the community. This element was not realised, which had a major influence on achieving the original goals of the quality improvement project. Sustainable and reliable financial structures are essential to realise and secure quality improvements within (long-term) care. Though, the nature of the political and subsequent financial climate is that they are subject to change [4].

All mechanisms and events (merge, funding and COVID-19) combined resulted into limited commitment from management to the original aims of the quality improvement project. This finding is also supported by van Haeften et al. (2015) [29], stating that insufficient commitment from managers and project leaders hampers the implementation process.

### Core finding 3 – Collaboration with new stakeholders

*In order to create an environment wherein persons with dementia are truly part of society it is important that the external stakeholders understand, support and share the concept. These are mainly new stakeholders within the long-term care environment, such as municipalities, architects and the local church. It is important that a dementia-friendly society is deployed from multiple actors – not just the nursing home – and that long-term complex collaborations are sought to collectively implement the innovations.*

The collaborations with external partners are the main focus of core finding 3, which is therefore mainly linked to the ‘Outer Setting’ – domain.

The construction of dementia-friendly communities requires the involvement of stakeholders at different levels, with a prime focus on the persons with dementia themselves and their informal caregivers [10, 21, 22]. Intersectoral collaborations of among others research institutions, local and regional governments, policy makers (from care organisations) and local businesses and organisations are crucial for successful realisation, as they have to potential to create a supportive environment and combine knowledge and resources [10, 21, 22, 37].

However, due to their varying backgrounds, we found that these collaborations can also pose challenges as some of the stakeholders are not familiar with the long-term care setting and/or lack knowledge about (persons with) dementia. Moreover, we found that the involvement of some of these stakeholders is partially dependent on the resources provided (e.g. municipalities), which poses challenges when seeking long-term sustainable collaborations. A finding that is supported by other studies, which state that stakeholder involvement is rather unpredictable and that insuffient time and funding hamper processes and outcomes in this regard [22].

### Core finding 4 – Shift of community members

*The transition to a dementia-friendly society means that spaces must be created where persons with dementia and local residents can meet. To do this well, it requires a focus not only on residents, but also on the people in the community. This means that local residents are shifting from external to internal stakeholders. This turnaround extends beyond the regular involvement of informal carers and volunteers within the nursing home.*

The community is the primary focus of core finding 4 and therefore falls under the ‘Outer Setting – domain’, especially because this core finding comprehends the necessary shift of community members from external stakeholders to inner stakeholders. A shift that is key when realising a dementia-friendly community.

Succesess achieved within this quality improvement project are the restaurant, the new nursing home and the open long-term care environment for residents with dementia. Though, changing the place of the nursing home within the community and the subsequent focus on the well-being of community members and their central role as main stakeholders in the change process was not realised. As a result, the shift towards a dementia-friendly society has not taken place. A finding that is among others reflected in the reduced response rates for the questionnaires for community members between 2018 and 2021.

The inclusion of community members as internal stakeholders, as is the aim of dementia-friendly societies, is challenging. Current studies on social networks of residents (with dementia) living in long-term care facilities, mainly focus on the ‘Caregiving triangle’ with only nursing staff, residents and their family members [16, 38, 39]. Furthermore, these caregiving triangles put a strong emphasis on the practical engagement of family members within daily care to support nursing staff and facilitate person-centred care for their relatives [31, 39, 40]. Though, to realize a dementia-friendly society, the role of community members should extend beyond task-oriented and requires a changed focus on social capital and involvement in long-term care. This ‘new’ way of involvement is an absolute necessity to live among each other instead of next to each other, both in- and outside the facility. More insight into strategies to truly include community members in the inner setting of a nursing home is much needed.

As described within the four core findings, some important elements that were part of the original project plan are not achieved, most importantly the lack of realization of a ‘community approach’. Remarkably, the management of the care organisation and other involved stakeholders within the quality improvement project are content with the eventual realisation of the project, even though the transcending aim has not been achieved. Being able to provide new nursing home apartments and more autonomy for residents in their physical movement are seen as important improvements in quality of care. Nevertheless, nursing staff, family members and community members were more critical about the safety of the residents and their involvement within the change.

Also, residents hardly visit the park area on their own and there is still a strong emphasis on group-activities, instead of individualised daily activities. However, these aspects were not found to be problematic by the management of the care organisation. This is mostly attributable to the shift in management and the change of vision over time from community-oriented in 2018 towards a focus on long-term institutionalised care in 2021.

### Reflection on methodological approach

To gain a broad view of the implementation process and the stakeholders involved, ethnographic thick description for quality improvement and safety according to Leslie et al. (2014) [13] was combined with the CFIR. The CFIR has been used regularly as a theoretical framework within long-term care studies and was combined with varying research methods [36, 41–43] and is especially well suitable to evaluate dementia-friendly initiatives [10]. We noticed that it is challenging to use both the CFIR and ethnographic approach alongside each other, as they have their origin in different research paradigms. The encountered challenges are reflected within our analyses approach. Initially, we qualitatively analysed the focus groups with the management team using data coding. Yet, this approach was not feasible to structure all the data collected in this paper. For this reason we made the decision to base our analyses of all the data collection on theme identification.

We did experience that using theme identification provided us with the possibility to gain an in-depth understanding of the involved mechanisms. Proces evaluations increase the chance of successful implementation of quality improvement projects, by having the ability to conduct a structured evaluation and provide insight into the nature and exposure and the experiences of those exposed [2]. Furthermore, using ethnography is notably valuable when studying unplanned and unexpected changes during implementation [12], e.g. global pandemics and corporate mergers.

Due to this structured evaluation, vital facilitating and hindering mechanisms for implementation can ultimately be indicated [2]. As ethnographic evaluations have the potential to support leaders during the implementation of quality improvement projects [44], more research on the complexity and the added value of ethnography would therefore be beneficial for future studies.

### Strengths and limitations

To the best of our knowledge, this is one of the very first evaluation studies of an extensive change process that aims to enhance the autonomy and freedom and integration of persons with dementia living in long-term care facilities. We found that using various research methods and valuing them as equal (according to the ethnographic approach) is very useful to study complex change processes. It supports researchers to gain a nuanced understanding of the social context in which the change process takes place [13] and provides insight into underyling mechanisms that other methods cannot [12]. As we used a variety of research methods, the research team often visited the nursing home and gained therefore substantial insight into the change process, which might be beneficial for future studies.

This study also has its weaknesses. An evaluation study was part of this research project. Though, due to the limited number of residents involved, it was not possible to test the differences before and after the project statistically. Moreover, due to the methodological decisions, not all interviews were audio recorded and transcribed verbatim. Though, in line with the foundation of ethnography, informal interviews are part of data collection and considered valuable for analyses [12]. Lastly, in this project we used the original CFIR [9] instead of the updated version based on user feedback [45] as the original framework was already used from the beginning of the project, which started a few years before the updated version was published.

When research is an important part of an implementation process and scientific institutes are collaboration partners within the research project, it is nearly inevitable that this dynamic has some kind of influence on the progress. To fully reflect on the implementation process, it is important to take the role of the researchers involved in this study into account. Within the collaboration agreement that was formed (as described within the methods section), it was established that Nivel carried the main responsibility for the project and was in the lead regarding the reports towards the funding organisation. Nivel was, therefore, the most dependent on sufficient progress of the project in order to be able to develop the deliverables. As some of these deliverables consisted of reports and articles regarding the last (and final) phase of the project, the time line of the process and organisation of data collection was regularly discussed during meetings with the care organisation. This external pressure from the researchers might have influenced the course and the advancement of the project and subsequent implementation process. Possibly resulting into a care organisation that stayed closer to the initial innovation. Furthermore, the care organisation potentially felt the need to realize some core components of the project that were otherwise neglected and meet the previously set deadlines.

## Conclusion

This study provides meaningful insight into the process of implementing a new long-term living concept for persons with dementia and is therefore an important contribution to the existing knowledge on the implementation of quality improvement projects within long-term institutionalised care. As nursing homes are part of the local community, it provides opportunities to collaborate on a dementia-friendly society. However, the change that is required (promoting freedom, residents’ autonomy and the redesign of care processes) is complex and is influenced by various mechanisms at different levels. Understanding these mechanisms is beneficial for other care organisation that want to implement a similar initiative.

### Supplementary Information

**Supplementary Material 1. **

**Supplementary Material 2. **

**Supplementary Material 3. **

## Data Availability

The datasets analysed during the current study are not publicly available due to privacy reasons, but are available from the corresponding author on reasonable request.

## Abbreviations

CFIR: Consolidated Framework for Implementation Research
PDCA: Plan Do Check Act
TDF: Theoretical Domains Framework
