# Supplementary material for: Implementing a new living concept for persons with dementia in long-term care: evaluation of a quality improvement process
Source: BMC Health Serv Res. 2024 Mar 7;24:306. doi: 10.1186/s12913-024-10765-y (PMC10921681; doi:10.1186/s12913-024-10765-y)
Supplement: Supplementary file 1 — Supplementary Material 1. [file 12913_2024_10765_MOESM1_ESM.docx]

**Additional file 1: Description of the original temporary building (qualitative observation)**

The care for people with dementia is situated in a dated temporary building on the grounds and includes two psychogeriatric units. The psychogeriatric ward consists of one unit on the ground floor, split into two sections, and provides dementia care to 28 residents in total. Both sections have their own living room, with the connecting individual rooms located in the surrounding corridors. The corridors are long and most corridors have a dead end. The location provides more than enough walking space for its residents. Several seating areas have been created and are decorated with older and ‘home-like’ objects. The ward is accessible through double automatic doors secured by a code. Both living rooms have a kitchen, seating area and multiple dinner tables. Both living rooms have access to two medium size fenced gardens with some seating areas and restricted walking space. All residents have their own individual room and bathroom. Residents have the opportunity to bring their own furniture and personal belongings from home.
