# Supplementary material for: Implementing a new living concept for persons with dementia in long-term care: evaluation of a quality improvement process
Source: BMC Health Serv Res. 2024 Mar 7;24:306. doi: 10.1186/s12913-024-10765-y (PMC10921681; doi:10.1186/s12913-024-10765-y)
Supplement: Supplementary file 2 — Supplementary Material 2. [file 12913_2024_10765_MOESM2_ESM.docx]

**Additional file 2: questions included in interview guide for focus groups with nursing staff**

*Focus group September 2019*

Physical activity

- When talking about physical activity on the wards. What comes to mind?
  - What do you consider as physical activity?
  - How is this implemented on the ward?
- What is your opinion on physical activity for people with dementia living in nursing homes?
  - What do you consider as most important for residents?
  - Is it feasible to achieve this?
  - What challenges do you face?
  - Do you have suggestions and/or solutions to overcome these challenges?
- How would the ideal situation look like when it comes to physical activity of residents on the wards?
  - What is needed to achieve this?
  - Who should be involved?
  - Do you see a role for professional caregivers?

Safety

- *When talking about safety on the wards. What comes to mind?*
  - *Do you consider the ward as safe?*
- *Do you ever face difficult decisions regarding the safety of residents?*
  - *Could you give an example?*
- *What considerations are taken into account during the decision-making regarding the safety of residents?*
  - *Whom are involved in these decisions?*
- *What is your general view on safety and freedom?*
  - *Could you come up with an example of a situation wherein you faced an (ethical) dilemma?*
  - *What do you consider as most important?*
  - *How do you handle (potential) dilemmas? What would the ideal situation look like for you when it comes to the safety and freedom of residents?*

Overarching

- What would be the ideal situation for you regarding safety and freedom of residents?
- Do you see a connection between safety, freedom and physical activity?
  - If so, how?
  - Could you give an example?
  - How do you handle these situations?

Experiences with/attitude towards change at [name care facility]

- What do you think of the (intended) changes at [name care facility], specifically:
  - The new building
  - The chapel
  - The grounds/park
- How will these changes affect residents do you think?
- How are you involved within the change process?
  - Was this sufficient?

*Focus group October 2021*

Icebreaker for introduction

We put a number of objects on the table such as toy scrap truck, walking stick, plant, face mask, stethoscope, board game, sausage roll / food, toy dolls. There are some items on the table. We ask you to briefly introduce yourself, name and function within [name care facility], and then choose 1 object on the table that you most associate with [name care facility]and explain why you associate it with [name care facility].

Experiences with/attitude towards the changes at [name care facility]

- Much has changed at [name care facility] in recent times. How did this go?
  - Are there factors that have positively or negatively influenced the changes at [name care facility]?
- What do you think of the changes at [name care facility], specifically:
  - The new building
  - The chapel
  - The grounds/park

The consequences of the changes at [name care facility] on residents

- How do you think the changes have affected residents?
  - Do residents walk more or less?
  - Do residents go out more or less?
  - Do you feel that residents feel more or less comfortable in their new environment? (welfare)
  - Do residents have more or less social contacts with their neighbours/family?
  - Do you see more or less residents walking compulsively/being restless?

The consequences of the merger on the changes at [name care facility]

- There has also been a merger between [name care organization 1] and [name care organization 2]. How did this go?
  - What do you think of this merger?
  - What do you notice in practice?
- In your opinion, has the merger influenced the (planned) changes at [name care facility]? Can you explain how or in what way?

The consequences of the changes at [name care facility] on the work (satisfaction) of care providers

- Has your way of working changed due to the changes at [name care facility]? Can you explain how or in what way?
  - How do you experience the new interpretation of your work?
  - Do you feel more comfortable or not?
- How were you involved within the change process?
  - Was this sufficient?
