# Supplementary material for: Implementing a new living concept for persons with dementia in long-term care: evaluation of a quality improvement process
Source: BMC Health Serv Res. 2024 Mar 7;24:306. doi: 10.1186/s12913-024-10765-y (PMC10921681; doi:10.1186/s12913-024-10765-y)
Supplement: Supplementary file 3 — Supplementary Material 3. [file 12913_2024_10765_MOESM3_ESM.docx]

**Additional file 3: questions included in interview guides from three focus groups with management**

*Focus group one*

Opening question

- What is your role within the quality improvement project at [name care facility]?

Intervention Unadapted

- Can you tell us about the context in which this nursing home was created?
- What are the core elements of the quality improvement project?
  - How are these designed?
- Who do you think are the most important parties with whom you have been in contact from the start?
  - What is your view on these collaborations?
- On which aspects has there been a lot of consultation/discussion?
  - What was ultimately done with these aspects?
  - What considerations have been made in this regard?
- What do you think characterizes the changes at [name care facility]?

Individuals involved/Inner setting

- How have residents and their relatives, local residents and care workers been involved in the change process and what are their views?
- How do you think the change process has gone so far?
  - Which aspects are you proud of?
  - What were the challenges?
  - How did you deal with these challenges?

Process

- In what way is/will be the change process monitored and evaluated?

*Focus group two*

Opening question

- What happened at [name care facility] the past year?

Outer setting

- What important choices did you have to make this year?
- What are the experiences regarding the collaborations with the municipality and the province in the past year? For example, the construction of the fishing pond.
- How has the COVID-19 outbreak affected the new construction and relocation?
  - Did this lead to changes in planning?
  - What measures have been taken to ensure that the renovation and relocation run as smoothly as possible?
  - How is contact with local residents maintained?
  - How is contact with family members of residents maintained?

Individuals involved/Inner setting

- In the past period, many people involved in the project left the organization or started a different position within [name care organization]
  - When did the various employees leave or were reassigned?
  - What impact does this have?
  - Who has taken over his/her role?
- Did the change of management influence the quality improvement project?
  - Were any changes made to the original set-up as a result of the merger?
- During the previous focus group, the importance of including healthcare workers in the project and changes was discussed in detail.
  - How has attention been paid to this in the past year?
  - Which events/elements did or did not take place?

Process

- The residents' move was postponed to the end of November.
  - What were the reasons for this?
  - How did the move go?
- What is your view on the change process so far?
  - Which aspects are you proud of?
  - What were the challenges?
  - How did you deal with these challenges?
- What challenges are there right now?
- What will the planning for the upcoming period be?

*Focus group three*

Opening question

What is the most important topic within [name care facility] at the moment?

Outer setting

- What is the current status of the renovation and the park? What does further planning look like at this point?
  - Park
  - Old buildings
  - Individual apartments? Social rent?
- During the last focus group it emerged that contact with local residents and family members has been limited over the past 1.5 years.
  - What is the current status?
  - What initiatives have there been?
  - How will this be continued?

Intervention adapted

- During the previous focus group it was stated that there was tension regarding the summer and the expectation that more residents would go outside. How did this go?
  - Do the residents actually go outside? If so, can a distinction be made which residents?
  - How is this integrated within the organization of care?
  - Do residents visit the park?
- During the previous focus group it emerged that the technology was not working optimally yet. What is the current status?
  - Do residents have living circles?
  - Can residents now enter the facility independently?

Individuals involved/Inner setting

- During the previous focus group, the importance of including healthcare workers in the changes was discussed, and in particular the challenges that still lie with regard to this subject.
  - How has attention been paid to this in the past year?
  - What are you facing?
  - What plans do you have for the future?
- During the previous focus group, it was mentioned that residents with dementia live together on the first floor, since they moved in cohort due to COVID. The plan was to have the residents live on multiple floors among residents without dementia. What is the current status of this intention?
  - How will this be further shaped in the future?

Process

- In your opinion, is the concept of [name care facility] still relevant and is it still supported by the organization?
- A number of elements have been previously mentioned by [name care organization]. To what extent are these elements still important elements for the new (merged) organization?
  - Connection with the neighborhood?
  - Emphasis on well-being and activities
  - Organizing supply from the Wmo
  - Collaboration with local middle class
- What is the current status of the implementation of the new vision and new way of working?
  - What is currently going well?
  - What are you facing?
  - What does the further planning look like to achieve implementation?
  - How will the new vision and way of working be safeguarded within the organization of care?

Overall

- How do you view the course of the process?
  - Which aspects are you proud of?
  - What were the challenges?
  - How did you deal with these challenges?
- What would you like to pass on to other organizations that want to go through a similar process?
- What are the points of attention for [name care facility] in the next two years?
